# Pedagogy collection: a new innovative teaching and outreach toolkit launched by *Access Microbiology*

**DOI:** 10.1099/acmi.0.000702

**Published:** 2023-10-09

**Authors:** Georgios Efthimiou, Sean Goodman, Bridget G. Kelly, Melissa M. Lacey

**Affiliations:** ^1^​ Centre for Biomedicine, Hull York Medical School, Hull, UK; ^2^​ University of Liverpool, Crown Street, Liverpool, Merseyside, UK; ^3^​ Dundalk Institute of Technology, Dublin Road, Dundalk, Co. Louth, Ireland; ^4^​ Sheffield Hallam University, Howard Street, Sheffield S1 1WB, UK

**Keywords:** innovative teaching, outreach, Pedagogy Collection, *Access Microbiology*

## Introduction

Our world is rapidly changing in many different ways; because of this education must also evolve. Educators need to provide students with the skills to be able to navigate these changes, as well as to use advances in technology, such as Artificial Intelligence, to their advantage. In 2016, it was estimated that 65 % of current students will be employed in jobs that do not yet exist so it is vital that graduates gain skills that allow them to thrive in the future, such as creativity, problem-solving and critical thinking [1].

Innovative teaching techniques, which use a variety of different ways to engage students in their learning [2], help develop these skills, which are essential for future employment and career development. Finding ways to engage students has always been a challenge for educators, but in the last 10 years, it has become more of an issue. In 2020, during the COVID-19 pandemic, lecturers quickly pivoted to online learning in a very short space of time and used a variety of strategies in the place of face-to-face learning. In microbiology higher education, many innovative teaching techniques were born out of necessity during that time [3, 4] and have continued to be used effectively afterwards. Outreach is increasingly becoming a demand of higher education institutions and many innovative teaching techniques can include a community dimension [5].

In this paper, we describe the Pedagogy Collection, an innovative teaching and outreach toolkit, which contains a useful database of all pedagogy papers published in *Access Microbiology* since 2019. This toolkit will help educators of microbiology and related disciplines in their innovative teaching and outreach endeavours.

## Types of pedagogy articles

The dissemination of creative teaching approaches and instructional methodologies is essential for the expansion and development of the academic community in the field of science education. The open access online resource of *Access Microbiology* has established a place for itself by presenting a vast selection of freely available pedagogical and outreach papers that meet the needs of teachers, researchers and students alike. The development of the Pedagogy Collection embedded within this resource will act as an easily accessible fountain of knowledge, providing a wide range of educational articles with the aim to transform the practice of microbiology teaching and learning.

*Access Microbiology* provides a platform for educators to share their innovative teaching strategies. These articles delve into unconventional approaches to microbiology education, encouraging readers to think beyond traditional methods and adapt their strategies to the evolving needs of students. This is often through methods to encourage active learning, gamification of microbiology and hands-on experiments to stimulate curiosity and participation in biological education. Pedagogical articles in *Access Microbiology* often spotlight case-based learning practices, either within curricula modules or as outreach. These publications weave real-life scenarios into microbiological concepts, fostering critical thinking and problem-solving skills among students and the public alike, while deepening their understanding of microbial science and its wider implications.

As technology continues to reshape education, *Access Microbiology* showcases articles that explore the integration of digital tools in microbiology teaching. From virtual labs and interactive simulations to online collaborative platforms, these articles highlight how technology can enhance engagement, innovate assessment and facilitate remote learning. Articles on promoting inclusivity and diversity in microbiology education also find a prominent place on the platform. These articles tackle topics such as culturally responsive teaching, gender equity in STEM, and the accommodation of diverse learning styles.

Finally, *Access Microbiology* recognizes the importance of continuous professional development for educators. The platform features articles that provide guidance on refining teaching skills, staying up to date with the latest pedagogical trends, and effectively managing classroom dynamics.

## Pedagogy collection: our plan

The Pedagogy Collection is a new useful toolkit for microbiology educators, which will contain all pedagogy and outreach papers published in *Access Microbiology* since it was launched in 2019. The Collection will be set up on a separate webpage, under the *Access Microbiology* page of the Microbiology Society website. This page will contain links to all published pedagogical papers, including games, workshops and surveys, making finding relevant articles easier for the user. Pedagogical papers can also be found by using keywords in the search bar of the microbiologyresearch.org website.

We aspire that this toolkit will help colleagues teaching microbiology or similar biomedical and biological disciplines to improve the quality of their teaching, making it more interesting and engaging for their students. In addition, this will help boost their professional development and career expectations, as evidence of improving curricula and increasing student satisfaction remains a crucial target for all Higher Education institutions. Moreover, having such a solid educational database will hopefully attract more microbiologists to the field of pedagogical research and writing, especially those on a Teaching and Scholarship job track. The Microbiology Society is very keen on supporting such efforts, having waived publication fees for pedagogy and outreach submissions in *Access Microbiology*, while a very handy step-by-step guide for budding pedagogy-friendly microbiologists was published in 2022 by Lacey and Efthimiou [6].

## Linking the pedagogy collection with our society’s Education and Outreach Network (EON)

The Education and Outreach Network (EON) is a group within the Microbiology Society that consists of national and international Microbiology Society members. Members of the network mirror the Society’s values, with a range of career stages from PhD students to established academic colleagues interested in educating students at all stages, as well as members of the public, about microbiology. The Network aims to (1) support all Society members to share ideas and best practice linked to education and outreach, (2) champion education and outreach research and related activities and (3) encourage connections and collaborations between microbiology educators. EON has strong links with *Access Microbiology,* as shown by the Teaching Symposium at the Microbiology Society Annual Conference 2023 being sponsored by the platform and two posters linked to the Teaching Symposium winning the *Access Microbiology* prize. Within EON’s aim of championing education and outreach research, the group has provided webinars on publishing pedagogy and outreach research, as well as highlighting to members of the Society that *Access Microbiology* is an excellent option for publishing pedagogy and outreach research.

## Conclusion

Overall, it has become clear that the Microbiology Society is keen on playing a leading role in supporting and promoting pedagogy and outreach scholarship in the UK and beyond, as it has been realized that teaching quality and public engagement are two factors that need to be allowed to blossom! By creating helpful structures such as the Pedagogy Collection and EON, we aspire to take this further, widen our network with new enthusiastic colleagues and ensure that this ambitious collaborative effort leads to fruitful outcomes in the near future.

## References

[1] Soffel J (2023). Ten 21st-century skills every student needs. https://www.weforum.org/agenda/2016/03/21st-century-skills-future-jobs-students.

[2] Kalyani D, Rajasekaran K (2018). Innovative teaching and learning. J App Adv Res.

[3] Joshi LT (2021). Using alternative teaching and learning approaches to deliver clinical microbiology during the COVID-19 pandemic. FEMS Microbiol Lett.

[4] Fahnert B (2021). Absence makes the mind grow stronger - educating in a pandemic and beyond. FEMS Microbiol Lett.

[5] Pincott H, Hughes M, Cummins T, Morse DJ (2023). Building blocks of biofilms - an engaging and hands-on microbiology outreach activity for school children and the general public. Access Microbiol.

[6] Lacey MM, Efthimiou G (2022). A guide to pedagogical research for scientists from a biological sciences background. Access Microbiol.

